# The Effect of Ketamine on the Immune System in Patients with Treatment-Resistant Depression

**DOI:** 10.3390/ijms26157500

**Published:** 2025-08-03

**Authors:** Łukasz P. Szałach, Klaudia Ciesielska-Figlon, Agnieszka Daca, Wiesław J. Cubała, Katarzyna A. Lisowska

**Affiliations:** 1 Department of Pathophysiology, Faculty of Medicine, Medical University of Gdańsk, 80-211 Gdańsk, Poland; lukasz.szalach@gumed.edu.pl (Ł.P.S.); klaudia.ciesielska-figlon@gumed.edu.pl (K.C.-F.); agnieszka.daca@gumed.edu.pl (A.D.); 2 Department of Psychiatry, Faculty of Medicine, Medical University of Gdańsk, 80-211 Gdańsk, Poland; wieslaw.cubala@gumed.edu.pl

**Keywords:** ketamine, treatment-resistant depression, T cells, cytokines, IL-8, inflammation

## Abstract

Treatment-resistant depression (TRD) is associated with immune dysregulation. Ketamine, a rapid-acting antidepressant, may exert effects via immunomodulation. The aim was to examine ketamine’s impact on immune markers in TRD, including T-cell subsets, cytokines, and in vitro T-cell responses. Eighteen TRD inpatients received 0.5 mg/kg iv ketamine. Blood was sampled at baseline, 4 h, and 24 h to analyze T-cell phenotypes (CD28, CD69, CD25, CD95, HLA-DR) and serum cytokines (IL-6, IL-8, IL-10, TNF-α, IL-1β, IL-12p70). In vitro, PBMCs from TRD patients and controls were exposed to low (185 ng/mL) and high (300 ng/mL) ketamine doses. Ketamine induced a transient increase in total T cells and CD4^+^CD25^+^ and CD4^+^CD28^+^ subsets at 4 h, followed by a reduction in CD4^+^ and an increase in CD8^+^ T cells at 24 h, decreasing the CD4^+^/CD8^+^ ratio. Activation markers (CD4^+^CD69^+^, CD4^+^HLA-DR^+^, CD8^+^CD25^+^, CD8^+^HLA-DR^+^) declined at 24 h. Serum IL-10 increased, IL-6 decreased, and IL-8 levels—initially elevated—showed a sustained reduction. In vitro, high-dose ketamine enhanced the proliferation of TRD CD4^+^ T cells and dose-dependent IL-8 and IL-6 secretion from activated cells. Ketamine induces rapid, transient immune changes in TRD, including reduced T-cell activation and cytokine modulation. A sustained IL-8 decrease suggests anti-inflammatory effects and potential as a treatment-response biomarker.

## 1. Introduction

Depression is a severe mental condition that may be classified as major depressive disorder (MDD) or depressive episode due to bipolar disorder (BD). Around 30% of patients with MDD do not achieve remission, even after trying several antidepressants and augmentation strategies [1]. Those patients are often classified as treatment-resistant. Treatment resistance is defined as an inadequate response to 2 or more antidepressant trials of adequate doses and duration [2]. Despite many available treatment methods, treatment-resistant depression (TRD) is a considerable challenge for mental health professionals. Discovering new pathomechanisms of depression and seeking novel anti-depressive agents with different mechanisms of action is necessary to help patients suffering from TRD.

Several reasons, including misdiagnosis, comorbidity, inadequate treatment, psychosocial factors, or lack of compliance, might cause treatment resistance. However, genetic, neurobiological, and immunological factors play an important role in the pathomechanism of TRD [3]. Stress-induced excessive cortisol release induces dysregulation of the HPA (hypothalamic-pituitary-adrenal) axis. Cortisol activates the transcription of proinflammatory cytokines in microglia [4]. It has been shown that in TRD, pro-inflammatory cytokines induce an increase in the reuptake and impairment of the synthesis and release of monoamine neurotransmitters [5]. Moreover, pro-inflammatory cytokines interfere with the synthesis of monoamine neurotransmitters through the alteration of kynurenine and tetrahydrobiopterin (BH4) enzymatic pathways, resulting in reduced BDNF synthesis [4]. All of those processes contribute to depression and impaired treatment responsiveness.

Ketamine, a non-competitive N-methyl-D-aspartate (NMDA) receptor antagonist, is a well-known, widely used anesthetic and analgesic drug. Esketamine, an S(+) enantiomer in the intranasal form, was approved by the Food and Drug Administration (FDA) in 2019 as a new-generation, rapidly acting antidepressant [6]. Ketamine is a safe, well-tolerated, and effective medication in the treatment of both unipolar and bipolar depression [7]. The rapid and robust antidepressant effects of ketamine, both in its intravenous form [8] and intranasal administration [9], have been extensively documented and investigated worldwide. Unlike traditional monoaminergic antidepressants, ketamine primarily modulates glutamatergic transmission. By inhibiting NMDA receptors on GABAergic interneurons, ketamine induces a surge in glutamate release, activating α-amino-3-hydroxy-5-methyl-4-isoxazolepropionic acid (AMPA) receptors. This triggers intracellular signaling cascades involving brain-derived neurotrophic factor (BDNF) and the mechanistic target of rapamycin (mTOR), which promote synaptogenesis and restore synaptic connectivity in prefrontal and limbic regions implicated in depressive pathology [10,11]. Additionally, ketamine may upregulate serotonin 5-HT_1_B receptor density in key brain areas, potentially enhancing serotonergic signaling [12]. It exhibits anti-inflammatory properties by reducing pro-inflammatory cytokines and modulating the kynurenine pathway, mechanisms increasingly recognized in the etiology of depression [13,14]. Moreover, some studies have suggested a potential role of the complement system in mediating ketamine’s antidepressant effect [15]. However, it is still not fully understood by which mechanisms ketamine reduces depressive symptoms in TRD patients.

The immunomodulatory effect of ketamine has been previously reported in several studies, highlighting its potential role in modulating immune system activity in the context of depression [15,16]. It has been proven that ketamine modulates cytokine levels in patients with depression [17]. Kiraly et al. [18] performed a study in which TRD patients, after 4 h since receiving intravenous (iv) ketamine, presented decreased serum levels of interleukin 6 (IL-6) and granulocyte colony-stimulating factor (G-CSF), along with IL-1α and interferon gamma-induced protein 10 (IP-10). Another randomized, double-blind control study showed a decrease in IL-6 and tumor necrosis factor-alpha (TNF-*α*) levels just after 40 min and was correlated with a lower Montgomery-Asberg Depression Rating Scale (MADRS) score [19]. This is the first clinical study to support a positive correlation between changes in serum cytokine levels after ketamine infusion and improvements in depressive symptoms in patients suffering from TRD.

We previously demonstrated that TRD patients differ immunologically from healthy individuals. They were characterized by, among other things, a lower percentage of CD4^+^CD25^+^ cells and high serum IL-8 levels [20]. This time, we hypothesize that ketamine infusion can modulate different parameters of the immune system of patients suffering from TRD, especially the activity of T cells and their ability to produce pro- and anti-inflammatory cytokines, which can reduce inflammation and thus affect the mental state of patients. Our study takes a broad approach to the immunomodulatory effect of ketamine, not only by measuring serum cytokine levels at one time point, but simultaneously studying T-cell responses, phenotype, and cytokine levels in the blood over time after ketamine administration. Moreover, we included in vitro experiments to compare ex vivo results with a more exclusive examination of the ketamine immunomodulatory effect on T-cell activity.

We designed a study to compare how patients’ selected immune parameters change 4 and 24 h after ketamine infusions. We analyzed the percentages of helper (CD4-positive) and cytotoxic (CD8-positive) T cells expressing CD28, CD69, CD25, CD95, and HLA-DR antigens essential for T-cell functioning. We also determined the serum levels of inflammatory cytokines: IL-12p70, TNF, IL-10, IL-6, IL-1β, and IL-8. Additionally, we performed in vitro experiments to investigate how lymphocytes of TRD patients react to different concentrations of ketamine. We analyzed how ketamine influences their surface phenotype, proliferation capacity, and ability to produce cytokines. Our results were compared with samples from healthy volunteers.

## 2. Results

The mean of MADRS before ketamine administration was 28.9 ± 12 (min. 14; max. 42) points, while at the end of the series of ketamine administration (after 4 weeks) MADRS mean was 19.3 ± 8.6 (min. 1; max. 37) points (Table 1). In every patient receiving ketamine, we observed a reduction in the MADRS scale compared to the base level, although only 5 patients achieved remission (remission was defined as the total MADRS score arriving at ≤10 points [21]), and 1 patient met the criteria for response.

Patients were administered a ketamine dose of 0.5 mg/kg body weight [22]. During ketamine administration, we did not observe any serious side effects that would lead to discontinuation of the treatment. Some patients experienced dizziness, drowsiness, and dissociative symptoms, which did not persist for more than a few minutes after the infusion was stopped. In a few patients, a transient, mild increase in blood pressure during ketamine infusion was observed [23].

### 2.1. T-Cell Subpopulations and Serum Cytokine Levels Before and After Ketamine Administration Ex Vivo

**Figure 1:**
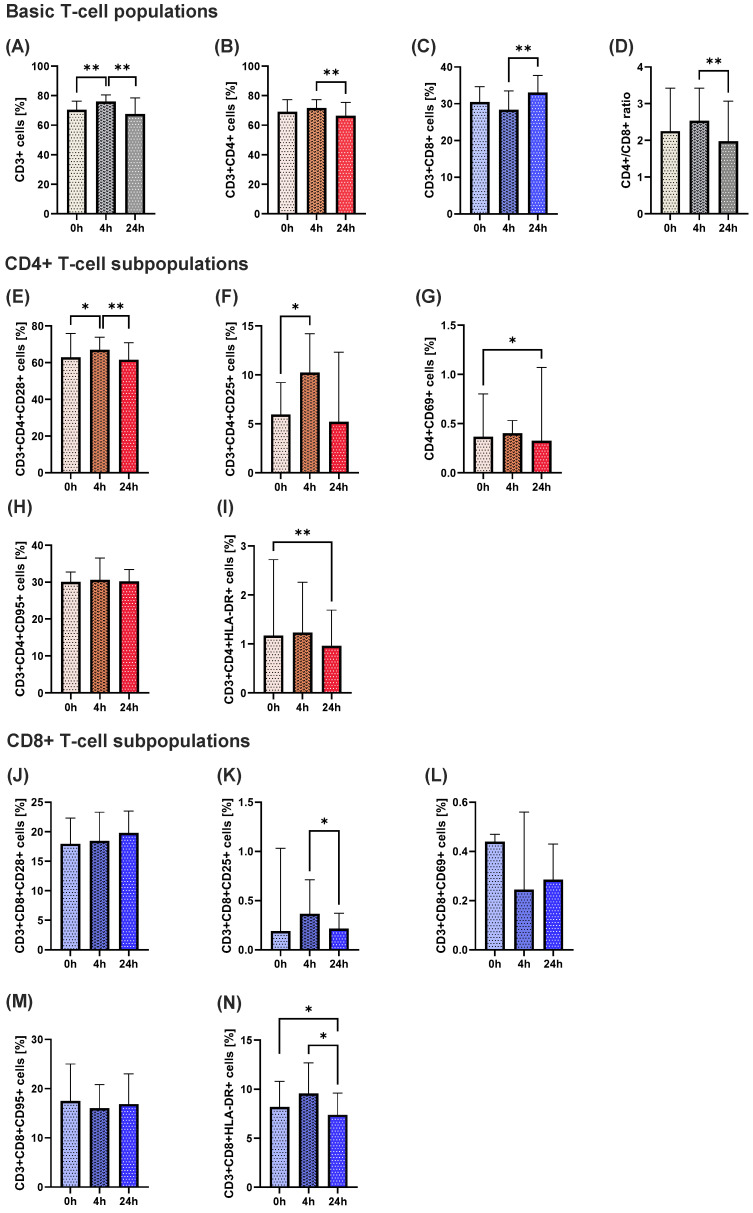
Comparison of percentage of CD3^+^ cells (**A**), CD3^+^CD4^+^ cells (**B**), CD3^+^CD8^+^ cells (**C**), CD4^+^/CD8^+^ ratio (**D**), CD3^+^CD4^+^CD28^+^ cells (**E**), CD3^+^CD4^+^CD25^+^ cells (**F**), CD3^+^CD4^+^CD69^+^ cells (**G**), CD3^+^CD4^+^CD95^+^ cells (**H**) CD3^+^CD4^+^HLA-DR cells (**I**), CD3^+^CD8^+^CD28^+^ cells (**J**), CD3^+^CD8^+^CD69^+^ cells (**K**), CD3^+^CD8^+^CD69^+^ cells (**L**), CD3^+^CD8^+^CD95^+^ cells (**M**), and CD3^+^CD8^+^HLA-DR^+^ cells (**N**) in TRD patients before 0 h, 4 h and 24 h after iv ketamine administration. The column bar graphs show the median value with confidence interval (CI). Wilcoxon test; * *p* < 0.05, ** *p* < 0.01.

We found a significant difference in the populations of CD3^+^ T cells between 4 h and 24 h after ketamine administration; at 4 h after ketamine administration, the percentage of CD3^+^ cells significantly rose, followed by a statistically significant drop after 24 h (Figure 1A). Similar tendencies were obtained for CD4^+^ T cells identified within the CD3 population, with a statistically significant fall after 24 h compared to 4 h (Figure 1B). On the other hand, a reversed effect was observed in the CD8^+^ T cells, where, after 24 h, the percentage of those cells increased significantly (Figure 1C). Finally, the CD4+/CD8^+^ ratio significantly decreased 24 h after vs. 4 h after ketamine administration (Figure 1D).

Differences between 4 h and 24 h after ketamine administration were also observed in the percentages of CD4^+^CD28^+^ cells (Figure 1E). After 4 h, the rate of cells increased, then significantly decreased after 24 h of ketamine administration. A rise in the percentage of CD4^+^CD25^+^ subpopulation was observed after 4 h as well (Figure 1F). The percentage of CD4^+^CD69^+^ (Figure 1G) and CD4^+^HLA-DR^+^ (Figure 1I) showed a significant decrease after 24 h compared to the starting point. No difference was seen in the percentage of CD4^+^CD95^+^ cells (Figure 1H). A significant reduction after 24 h was identified in the percentage of CD8^+^CD25^+^ (Figure 1K) and CD8^+^HLA-DR^+^ cells (Figure 1N) compared with 4 h. The percentage of CD8^+^HLA-DR^+^ cells was also lower compared with 0 h (Figure 1N). No difference was seen in the percentage of CD*^+^CD28^+^ (Figure 1J), CD8^+^CD69^+^ (Figure 1L), or CD8^+^CD95^+^ cells (Figure 1M).

**Figure 2:**
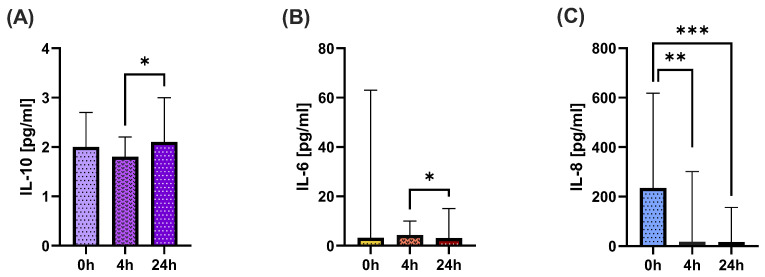
Comparison of serum concentration of IL-10 (**A**), IL-6 (**B**), and IL-8 (**C**) in TRD patients before 0 h, 4 h and 24 h after iv ketamine administration. The column bar graphs show the median value with CI. Wilcoxon test; * *p* < 0.05, ** *p* < 0.01, *** *p* < 0.001.

In the case of cytokines, serum IL-10 concentrations after 24 h compared to 4 h levels after ketamine significantly increased (Figure 2A). There were significant differences in serum IL-6 levels, in a similar manner of transient, although non-significant elevation after 4 h and following, a statistically significant decrease in level 24 h vs. 4 h after ketamine administration (Figure 2B). The levels of IL-1β, TNF, and IL-12p70 were close to 0 throughout the entire therapy, and therefore, were not included in Figure 2.

The most interesting findings were changes in IL-8 levels. Recently, we have shown that the TRD patients, who later received ketamine in vitro and were mostly included in this study, had higher serum IL-8 than healthy people—serum IL-8 levels above 19.55 pg/mL were associated with a 10.26 likelihood ratio of developing TRD [18]. Initially, very high levels of IL-8 before ketamine administration were observed, and rapidly and significantly dropped 4 h after ketamine administration, and with even stronger statistical significance 24 h after (Figure 2C).

### 2.2. IL-8 Levels After Following Ketamine Administrations

**Figure 3:**
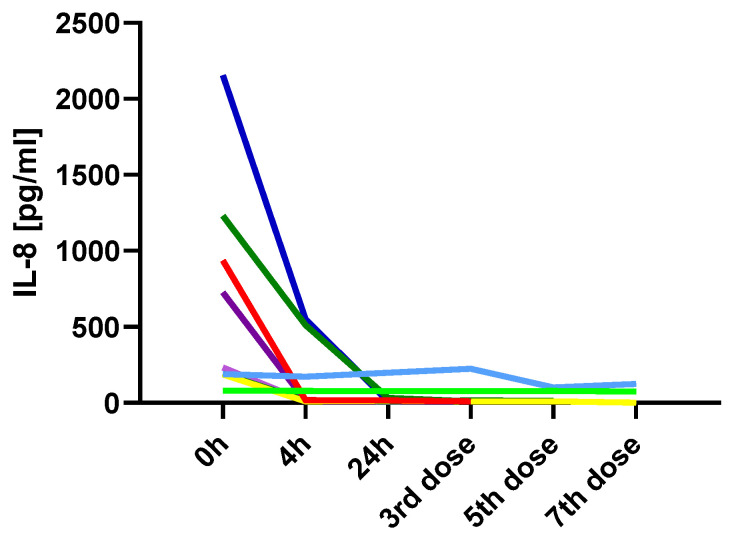
Serum IL-8 concentration during ketamine treatment in 9 TRD patients. Each colored line represents one of the patients.

Encouraged by the evident differences in serum IL-8 levels observed at 4 and 24 h after the first ketamine administration, we aimed to investigate whether this trend would remain over the following weeks of the study. Due to organizational constraints, we were able to collect blood samples at later stages of the study from only a limited number of participants. Blood samples were collected 24 h after the 3rd, 5th, and 7th (odd-numbered) ketamine infusions, at weekly intervals. Although the limited sample size precluded a reliable statistical analysis, reduced IL-8 levels persisted during the following weeks of treatment (Figure 3).

### 2.3. Correlations Between Immunological and Clinical Parameters

**Figure 4:**
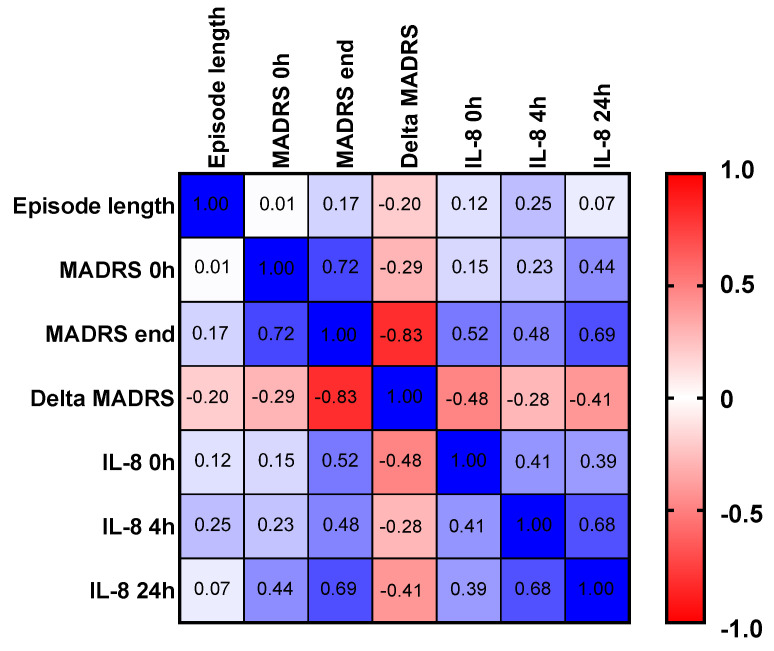
Heat map illustrating Spearman’s rank-order correlation (*r*) between episode length, MADRS scores at baseline and study endpoint, and IL-8 concentrations at various time points following ketamine administration. The color gradient reflects the strength and direction of correlations, with warm colors indicating positive correlations and cool colors representing negative correlations. The given r values are significant.

Spearman’s rank-order correlation analysis revealed several statistically significant associations (Figure 4). First, a strong positive correlation was observed between baseline and endpoint MADRS scores (*r* = 0.72, *p* = 0.001). Furthermore, a statistically significant positive correlation was found between endpoint MADRS scores and IL-8 concentrations measured before treatment (*r* = 0.52, *p* = 0.033) and 24 h (*r* = 0.69, *p* = 0.003) after the first ketamine administration.

### 2.4. T-Cell Activity and Cytokine Levels After Cell Activation in the Presence of Different Concentrations of Ketamine in Vitro

**Figure 5:**
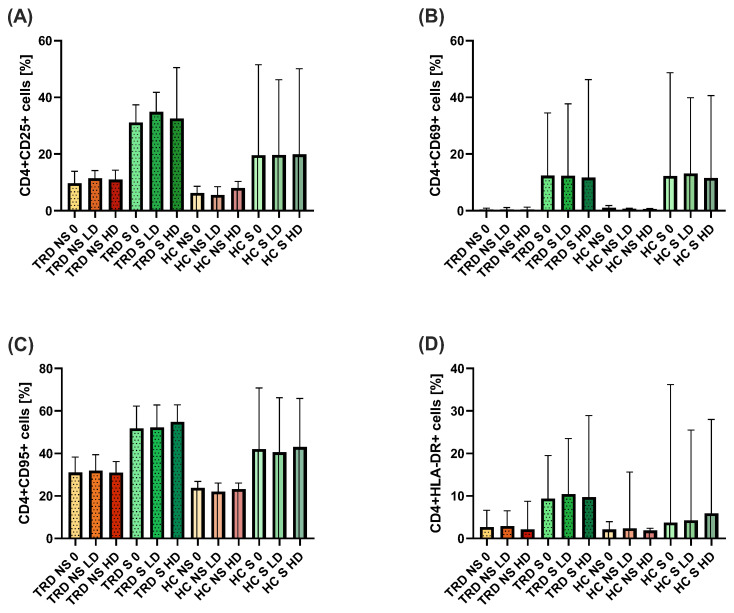

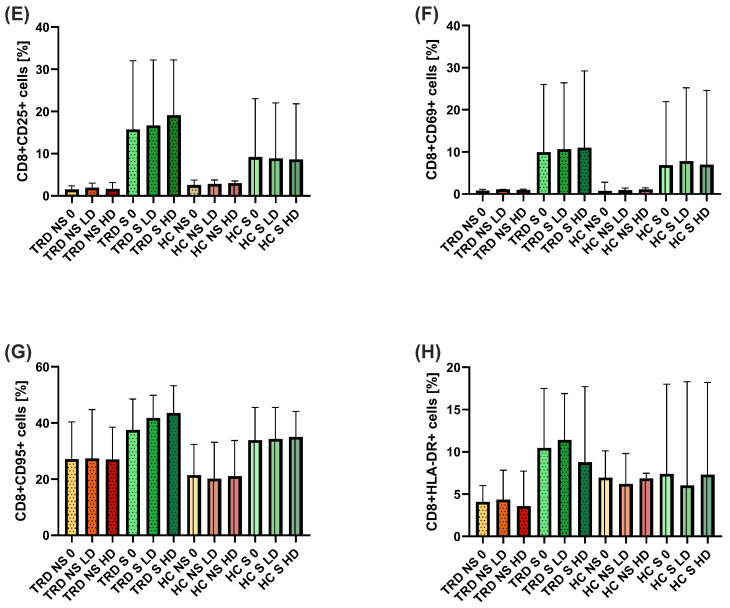
Comparison of the percentage of proliferating CD4^+^CD25^+^ cells (**A**), CD4^+^CD69^+^ cells (**B**), CD4^+^CD95^+^ cells (**C**), CD4^+^HLA-DR^+^ cells (**D**), as well as CD8^+^CD25^+^ cells (**E**), CD8^+^CD69^+^ cells (**F**), CD8^+^CD95^+^ cells (**G**), and CD8^+^HLA-DR^+^ cells (**H**). Cells of healthy individuals (HC) and TRD patients were stimulated with anti-CD3 (S) in the presence of 185 ng/mL (low doses—LD) or 350 ng/mL (HD—high dose) ketamine or without it (0) for 72 h. Non-stimulated (NS) cells were also incubated with different doses of ketamine. The column bar graphs show the median value with CI.

**Figure 6:**
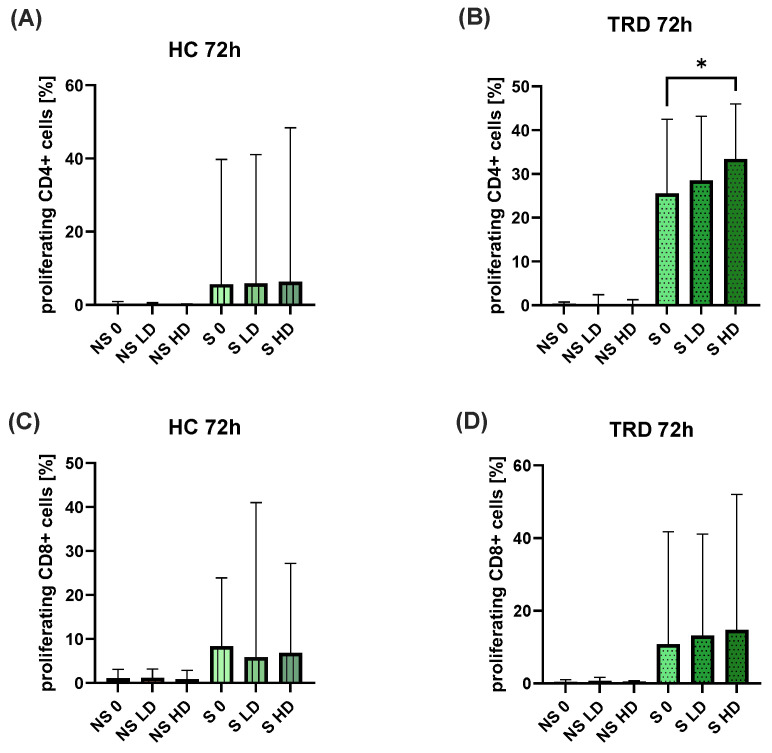
Comparison of the percentage of proliferating CD4^+^ and CD8^+^ cells from healthy controls (**A**,**C**) and TRD patients (**B**,**D**). TRD—patients, HC—healthy control, NS—non-stimulated, S—stimulated, 0—without ketamine, LD—low dose of ketamine, HD—high dose of ketamine. The column bar graphs show the median value with CI. ANOVA Friedman with Dunn’s multiple comparisons test, * *p* < 0.05.

We have found no difference in the percentages of CD4^+^ or CD8^+^ cells with expression of CD25, CD69, CD95, or HLA-DR antigens, regardless of the dose of ketamine (Figure 5A–H). The only statistically significant difference was observed in the percentage of proliferating CD4^+^ cells in TRD patients in the presence of HD of ketamine compared to stimulated cells without ketamine (Figure 6B). No other significant alterations in lymphocyte proliferation were observed (Figure 6A,C,D). Comparisons across varying ketamine concentrations (in both sub-groups of stimulated and non-stimulated cells) and between patient and healthy control groups showed no statistically significant differences.

**Figure 7:**
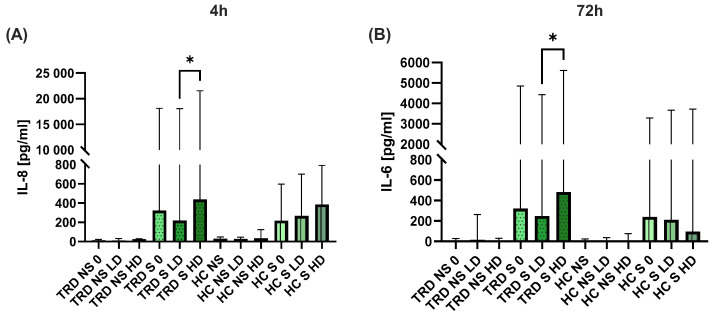
Supernatant concentrations of IL-8 (**A**) and IL-6 (**B**) after incubation with ketamine. TRD-patients, HC—healthy control, NS—non-stimulated, S—stimulated, 0—without ketamine, LD—low dose of ketamine, HD—high dose of ketamine. The column bar graphs show the median value with CI. ANOVA Friedman with Dunn’s multiple comparisons test, * *p* < 0.05.

As to cytokines, changes were observed only in IL-8 (Figure 7A) and IL-6 (Figure 7B). Levels of IL-8 were lower in the presence of 185 ng/mL (LD) ketamine compared to 350 ng/mL (HD). Similarly, levels of IL-6 were lower in the presence of LD ketamine than HD ketamine.

## 3. Discussion

Our study investigated immunological changes following ketamine administration in patients with treatment-resistant depression (TRD), using both ex vivo and in vitro models. In our previous study, we compared the immunological profile of TRD patients, who later received ketamine, to healthy volunteers. We found that patients with TRD exhibited a reduced percentage of CD4^+^CD25^+^, CD8^+^CD95^+^ cells; additionally, they showed lower serum levels of IL-12p70 and TNF-α, and highly elevated IL-8 levels compared to healthy controls [20]. Now, we observed dynamic alterations in T-cell subpopulations and cytokine profiles in the early period after a single ketamine infusion. These findings provide further evidence that the immunomodulatory effects of ketamine may contribute to its rapid antidepressant properties [24]. However, discussion of the effects of ketamine on the immune system is difficult. While there are isolated reports of its effects on serum levels of various cytokines [18,19], little is known about its effects on immune cells.

A transient activation of T lymphocytes was observed shortly after ketamine administration. Specifically, the percentage of total CD3^+^ T cells significantly increased 4 h after infusion, followed by a notable decrease 24 h post-administration. A similar pattern was found in CD4^+^ helper cells that play a central role in coordinating immune responses [25], while CD8^+^ cytotoxic T cells exhibited a reverse trend, with a delayed increase observed 24 h post-infusion. This divergence between CD4^+^ and CD8^+^ cells resulted in a decreased CD4^+^/CD8^+^ ratio at 24 h. The ratio is commonly used as an indicator of immune system homeostasis that is sensitive to stress and immunosuppressive agents [26]. Our results suggest a short-term shift in immune balance, with a biphasic response of initial activation followed by suppression of the immune system, possibly reflecting a state of temporary immune reorganization following ketamine administration.

Additional analyses of activation markers on CD4^+^ and CD8^+^ subsets supported this transient activation hypothesis. The percentages of CD4^+^CD28^+^ and CD4^+^CD25^+^ populations increased at 4 h, consistent with early T-cell activation, followed by a return to the initial level after 24 h. A similar observation was made for CD8^+^HLA-DR^+^ cells. Early activation marker CD69, a key T-cell activation marker, also showed a significant reduction at 24 h in CD4^+^ cells. These changes indicate that ketamine may initially trigger activation of helper and cytotoxic T cells, followed by a down-regulation of activation markers, potentially indicating an anti-inflammatory rebound or restoration of homeostasis.

The cytokine profile reinforces this interpretation. IL-6, a pro-inflammatory cytokine commonly elevated in depression, showed a significant reduction 24 h post-ketamine administration after a transient increase 4 h after ketamine. In parallel, levels of the anti-inflammatory cytokine IL-10 decreased after 4 h and then significantly increased 24 h later, supporting a transient shift toward a regulatory or resolving immune response. No changes were observed in other cytokines associated with inflammation, including TNF or IL-1β.

What is striking is that most of these ex vivo parameters, both blood T-cell subpopulations and serum cytokines, are transient shortly after ketamine administration. Park et al. [27], who also observed transient changes in serum IL-6 in MDD and BD patients, suggested that this could be a non-specific stress response since similar observations are seen after saline administration in patients who were undergoing coronary artery bypass graft surgery [28]. However, if this were true, this change could apply to all parameters, not just selected ones. It is therefore possible that, as suggested above, ketamine administration leads to the mobilization or transient activation of immune cells.

The only variable that not only changed shortly after ketamine administration but also remained at the achieved level is IL-8. TRD patients showed markedly elevated baseline IL-8 before ketamine administration, which significantly decreased both 4 and 24 h post-infusion. Moreover, continuous monitoring of IL-8 levels in several patients throughout the therapy showed that this decrease was maintained. Interleukin-8 (IL-8), also known as CXCL8, is a chemokine that plays a crucial role in the recruitment and activation of neutrophils during inflammatory responses. Elevated levels of IL-8 have been observed in various chronic inflammatory conditions, including major depressive disorder (MDD) [29]. The role of interleukin-8 as a marker of inflammation-related depression and predictor of treatment outcome has been previously described [30]. In our previous study, we found that in TRD patients, high serum IL-8 levels were associated with a 10.26 likelihood ratio of developing TRD [20]. The profound suppression of IL-8 may indicate a strong anti-inflammatory effect of ketamine, particularly on the innate immune system.

A strong positive correlation between endpoint MADRS scores and IL-8 levels measured before treatment and 24 h after the initial ketamine administration suggests a potential link between the inflammatory response and the clinical outcome. Specifically, higher IL-8 levels at 24 h were associated with greater residual depressive symptoms. None of the other immunological parameters showed such associations with treatment outcomes. Our findings strengthen the potential of IL-8 as a biomarker of inflammation-related depression and predictor of ketamine treatment response.

Our in vitro findings from cell cultures investigating the direct effects of ketamine on lymphocyte function were notably different and more limited in scope. In the cell culture model, ketamine did not significantly alter the expression of key activation markers (CD25, CD69, CD95, HLA-DR) on CD4^+^ or CD8^+^ T cells, regardless of the concentration used. This contrasts with our ex vivo data. The only statistically significant in vitro findings were an increase in CD4^+^ cell proliferation and increased levels of IL-6 and IL-8 in TRD patients’ stimulated cells with high ketamine concentrations compared to lower concentrations, suggesting a potential activating effect on T cells under strong exposure to the medication.

Studies investigating the effects of ketamine on human lymphocytes in vitro remain limited. In one study focused on a topic, although performed in a population of patients with gastric cancer, their PBMCs were incubated for 24 h with different concentrations of ketamine (25, 50, and 100 μm, which corresponds to 6.9, 13.7, and 27.4 ng/mL). The ratio of CD4^+^/CD8^+^ cells, as well as the percentage of Tregs, was significantly increased in the presence of rising concentrations of ketamine [31]. Their in vitro results somewhat overlap with our ex vivo results—in TRD patients, a transient increase in CD4^+^/CD8^+^ was observed 4 h after ketamine administration. Another study investigating the effects of ketamine on the proliferation of neural progenitor cells coming from healthy human-derived cells demonstrated that these cells exhibited a statistically significant increase in proliferation after 24 h of incubation. This effect was most pronounced at a ketamine concentration of 0.5 mM, which corresponds to approximately 137 ng/mL, and was inversely proportional to increasing ketamine concentrations [32]. In our study, proliferation of CD4^+^ cells was increased only in TRD patients and only in high doses of ketamine (350 ng/mL). These results show that the effects of ketamine are highly dependent on the dose, the type of cells stimulated, and even the cell donor.

Findings concerning cytokine production in the presence of ketamine partially align with the results of Kawasaki et al. [33]. In that study, lipopolysaccharide (LPS)-stimulated healthy human peripheral blood cells were incubated in the presence of ketamine. At a concentration of 100 ng/mL, ketamine was shown to directly inhibit the synthesis of TNF-α, IL-6, and IL-8 [33]. In our study, the production of IL-6 and IL-8 by lymphocytes from TRD patients stimulated in the presence of low-dose (185 ng/mL) ketamine revealed a downward trend. However, a high dose of ketamine significantly increased levels of those cytokines compared to the low-dose. Once again, these results show that the effect of ketamine is dose-dependent and cell donor-dependent.

The divergence between ex vivo and in vitro results indicates that the immunomodulatory effects of ketamine observed in vivo are likely not mediated only through direct action on lymphocytes but rather involve much more complex systemic and context-dependent mechanisms. These may include modulation of neuroimmune signaling via the HPA axis, autonomic nervous system, brain-derived neurotrophic factors, indirect effects through other immune cells such as monocytes or microglia, and cytokines and other substances secreted by them, changes in circulating metabolites such as kynurenines, as well as the permeability of the blood-brain barrier.

Nonetheless, some alignment was found at the cytokine level. In vitro, high-dose ketamine led to reduced IL-8 secretion after 4 h of incubation in stimulated lymphocyte cultures from TRD patients, echoing the robust and rapid drop in serum IL-8 levels seen ex vivo. This supports the theory that IL-8 suppression may represent a direct immunological target of ketamine, particularly in immune-activated states. Additionally, in vitro reductions in IL-6 at lower ketamine concentrations (observed only in TRD-derived cells) also parallel the ex vivo decrease in circulating IL-6 levels 24 h post-infusion.

Our study has several limitations. Due to the inability to administer ketamine to healthy individuals, we were not able to compare the ex vivo effects of ketamine in healthy subjects versus those suffering from TRD. We also could not examine the pure effect of ketamine, as all patients continued their baseline oral medications, which varied between individuals. Furthermore, strict regulations surrounding ketamine administration limited the size of the study group. As a result, we were unable to divide patients into subgroups based on sex, diagnosis (MDD vs. BD), or comorbidities such as anxiety disorders, to obtain statistically meaningful results in those groups.

Further research in this area—particularly studies examining the immunological profile of patients over 4 weeks of ketamine treatment and correlating it with psychometric data—would significantly strengthen conclusions regarding ketamine’s immunomodulatory effects as a potential antidepressant mechanism of action. Understanding how ketamine’s effects on the immune system contribute to clinical improvement in patients with treatment-resistant depression—and identifying the immunological profiles of those most likely to benefit—could enhance the ability to match patients with this treatment, ultimately increasing the likelihood of therapeutic response across a broader population.

## 4. Materials and Methods

### 4.1. Study Group

The study groups consisted of 18 inpatients diagnosed with depression in the course of MDD or BD without psychotic features (Table 1). The study population includes subjects enrolled in a naturalistic observational registry protocol for intravenous ketamine treatment in TRD: A Naturalistic Study of Ketamine for Treatment-Resistant Mood Disorders (GDKet) (NCT04226963). Clinicians examined subjects using the Mini-International Neuropsychiatric Interview (MINI) to verify the diagnosis using the Diagnostic and Statistical Manual of Mental Disorders (DSM-5) criteria and with the Montgomery–Asberg Depression Rating Scale (MADRS).

Only patients meeting the criteria for a TRD diagnosis were eligible for the study. A patient was defined responder at the end of the study if the improvement from the baseline total MADRS score was at least a 50% reduction of points and was not qualified as remitted. Remission was defined as the total MADRS score arriving at ≤10 points [21]. All participants were described as having treatment resistance for the current episode, assessed by the Massachusetts General Hospital Antidepressant Treatment Response Questionnaire (ATRQ).

The exclusion criteria from the research were uncontrolled arterial hypertension, unstable coronary artery disease, increased intracranial pressure, acute and chronic infectious diseases, inflammatory, autoimmune, and metabolic diseases, and neoplastic diseases. In addition, mental disorders could not be present in the control group. Only medically stable, able to communicate, and provide consent adult inpatients were enrolled in the study.

All subjects gave written informed consent to participate in the study. After the procedures had been fully explained, written consent was obtained from each participant. The Independent Bioethics Committee for Scientific Research approved the study (consent No. NKBBN/398/2017 received 12 October 2017). We performed all the experiments following the relevant guidelines and regulations.

The in vitro experiments were performed on 4 TRD patients from 18 who were qualified for this study and 4 healthy people, 2 women and 2 men in each group.

The tested material was up to 20 mL of peripheral venous blood collected into EDTA (ethylenediaminetetraacetic acid) tubes. In addition, 5 mL of blood was collected into anticoagulant-free tubes to collect serum to assess concentrations of cytokines. We stored serum samples at −80 °C.

### 4.2. Study Design

**Figure 8:**
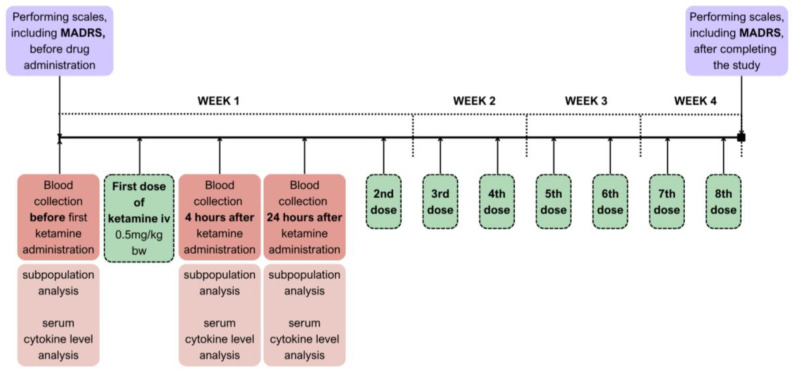
Timeline of the study procedure showing time points of intravenous ketamine administration, blood sample collection, and MADRS assessments.

The study followed an observational design; all patients continued their baseline psychotropic treatment (Figure 8). The therapeutic intervention included intravenous ketamine infusion, administered at a dose of 0.5 mg/kg based on the actual body weight of the patient and given over 40 min. The preparation used for infusions was Ketalar 50 (ketamine hydrochloride) 50 mg/mL; one vial contained 10 mL. The managing psychiatrist monitored safety before, during, and after the infusion every 15 min to 1.5 h, including periodic assessment of vital signs (heart rate, body temperature, respiration rate, blood pressure, and oxygen saturation). Safety monitoring also included the Brief Psychiatric Rating Scale (BPRS) and Clinician-Administered Dissociative States Scale (CADSS) at baseline and one hour after the infusion.

Peripheral venous blood was collected three times from each patient, before ketamine infusion, 4 and 24 h after ketamine administration.

### 4.3. Determination of T Cell Subpopulations Ex Vivo

A total of 100 mL of blood samples were transferred for staining with monoclonal antibodies and red blood cell (RBC) lysis. RBCs were lysed with buffer containing 0.8% NH_4_Cl and 0.1% KHCO_3_. Cells were then washed with PBS (phosphate-buffered saline) buffer and stained with FITC-conjugated anti-CD3 or anti-CD95, PE-Cy5-conjugated anti-CD4, PE-conjugated anti-CD28, anti-CD25, anti-CD69, or anti-HLA-DR, APC-H7-conjugated anti-CD8 (BD Pharmingen, San Diego, CA, USA) for 30 min at 4 °C in the dark. After this time, cells were washed with PBS and suspended in 200 mL of suitable buffer for flow cytometric analysis using the FACSVerse instrument (Becton Dickinson, Franklin Lakes, NJ, USA).

### 4.4. Cytokine Measurement in Serum and Plasma Samples

Cytometric Bead Array (CBA) Human Inflammatory Cytokines Kit (BD Biosciences, San Jose, CA, USA) was used according to the manufacturer’s protocol to determine the level of IL12p70, TNF, IL-10, IL-6, IL-1β, and IL-8 in the serum samples from TRD patients and culture supernatants. Then, quantitative cytometric fluorescence analysis was performed. The detection range for all measured cytokines was between 20 and 5000 pg/mL. The concentrations were analyzed using X = log(X) transformation and non-linear regression, with least squares regression fitting using GraphPad Prism version 9 (GraphPad Software, Boston, MA, USA).

### 4.5. PBMC Isolation and Stimulation

A total of 15 mL of peripheral venous blood was collected from 4 healthy volunteers and 4 patients with depression (2 diagnosed with BD and 2 with MDD) in tubes containing EDTA. Then, peripheral blood mononuclear cells (PBMC) were isolated by centrifugation on Histopaque™ gradient (Sigma Aldrich Inc., Saint Louis, MO, USA). PBMCs were stained with Violet Proliferation Dye 450 (VPD450) (Becton Dickinson, Franklin Lakes, NJ, USA) for 12 min in the dark at 37 °C according to the manufacturer’s protocol. Cells were then washed in phosphate-buffered saline (PBS) (EURx, Gdańsk, Poland) and resuspended in a complete culture medium (RPMI 1640 supplemented with 10% fetal bovine serum, 2 mm L-glutamine, 100 U/mL penicillin, and 100 μg/mL streptomycin) at a concentration of 1 million cells per 1 mL medium. Cells were divided into three different subgroups according to the solution of ketamine in which they were incubated: 0 mmol/L (control), 185 ng/mL (low doses—LD), and 350 ng/mL (HD—high dose). The concentration of ketamine in the culture was selected based on reports of blood ketamine levels in individuals receiving the drug at a dose of 0.5 mg/kg body weight [22].

The cells were incubated with an immobilized (tissue-culture plate-bound) monoclonal anti-CD3 antibody (BD Pharmingen, San Diego, CA, USA) with the addition of ketamine solutions in standard culture conditions (5% CO_2_, 100% humidity at 37 °C) for three days.

Stimulated cells were collected after 4 h, 24 h, and 72 h and stained with the following antibodies: FITC-conjugated anti-CD95 and anti-CD25, PE-Cy5-conjugated anti-CD4, PE-conjugated anti-CD28, anti-CD69, and anti-HLA-DR, and APC-H7-conjugated anti-CD8 (BD Pharmingen, San Diego, CA, USA). Cells were also stained with PE-conjugated annexin V or 7-aminoactinomycin D (7-AAD), according to the manufacturer’s protocol (BD Pharmingen, San Diego, CA, USA), and analyzed with flow cytometry.

### 4.6. Analysis and Statistics

**Figure 9:**
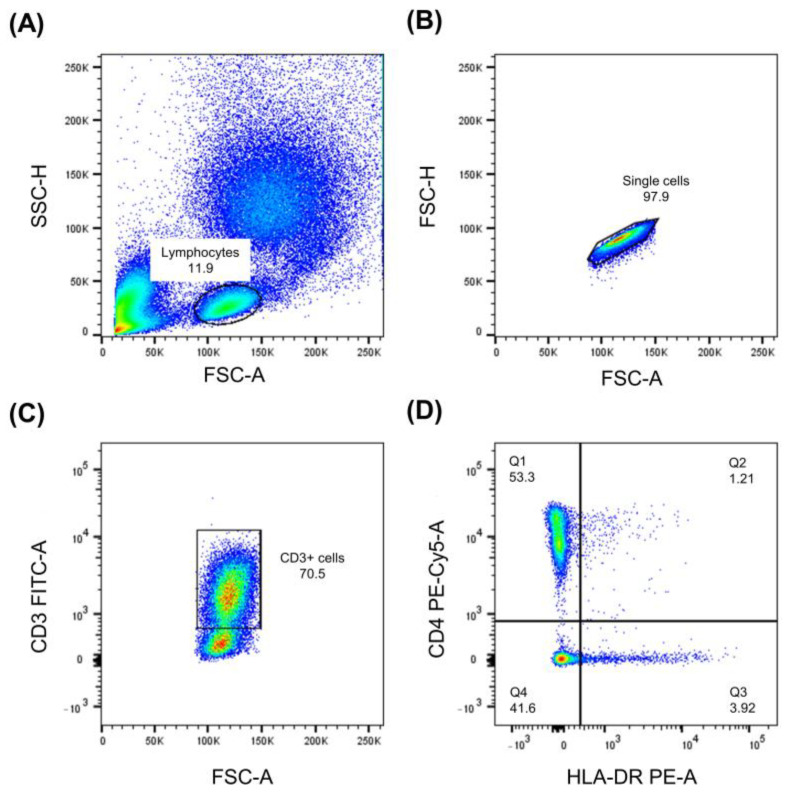
Analysis of lymphocytes ex vivo. Lymphocytes were selected based on forward and side scatter characteristics (**A**); only single cells were included in the analysis (**B**). T cells were identified based on their positivity for the CD3 antigen (**C**). Helper T cells were identified based on the expression of CD4 antigen, and finally, activation antigens, e.g., CD25 antigen, were identified (**D**). The figures are presented as density plots that show cell distribution with areas of high cell concentration appearing as denser, red colored.

Twenty thousand events corresponding to light scatter characteristics of viable lymphocytes were acquired from each sample to analyze lymphocyte subpopulations. First, lymphocytes were selected based on forward and side scatter characteristics (Figure 9A); only single cells were included in the analysis (Figure 9B). Then, T cells were identified based on their positivity for the CD3 antigen (Figure 9C). Next, helper T cells were recognized based on the expression of CD4 antigen, and cytotoxic T cells based on CD4 and CD8 expression. Finally, subpopulations expressing different activation antigens, e.g., CD25 antigen, were identified (Figure 9D). The cytometric data were analyzed using the FlowJo version 10 software (Beckton Dickinson, Franklin Lakes, NJ, USA).

**Figure 10:**
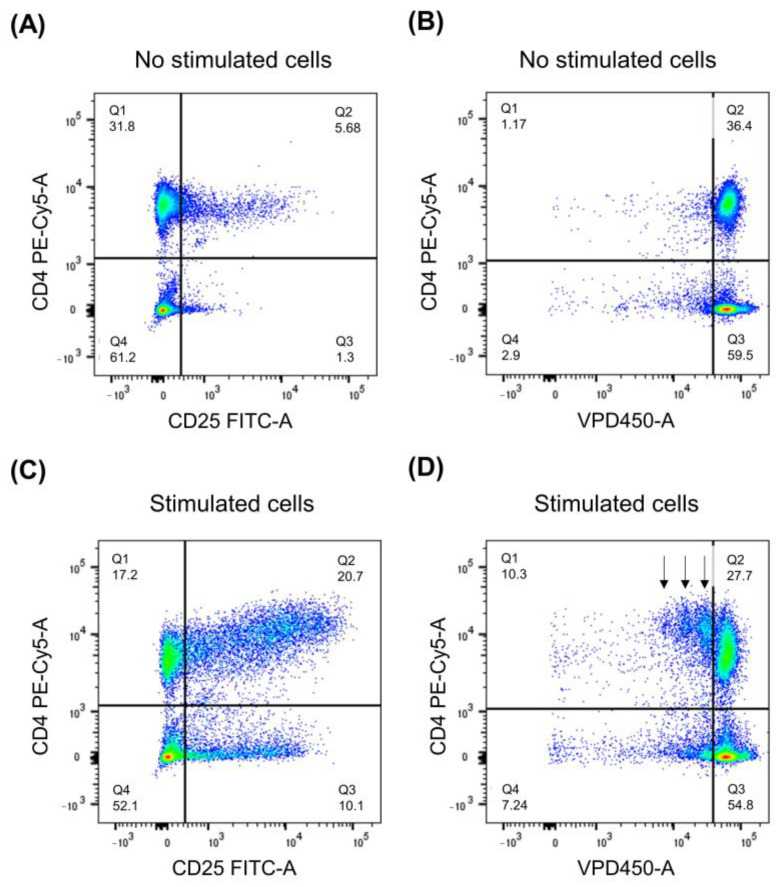
Analysis of phenotype and proliferation of lymphocytes in vitro. Non-stimulated (**A**,**B**) and stimulated (**C**,**D**) lymphocytes were identified based on the expression of CD4 antigen. Expression of activation antigens, e.g., CD25, was analyzed (**A**,**C**). VPD450 dye was used to analyze proliferating cells (**B**,**D**), which are marked with arrows (**D**). The figures are presented as density plots that show cell distribution with areas of high cell concentration appearing as denser, red colored.

To analyze lymphocyte proliferation patterns and cell phenotype after stimulation, thirty thousand events corresponding to lymphocytes were acquired from each sample. The expression of activation antigens, e.g., CD25, was identified on non-stimulated (Figure 10A) and stimulated (Figure 10C) lymphocytes. We used the dividing cell tracking (DCT) method to track cell proliferation using VPD450 (Figure 10B,D). After cleavage by cellular esterases within viable cells, the dye became fluorescent and covalently bound to proteins within the cells. As viable cells divided, the VPD450 dye was distributed uniformly between daughter cells, so each daughter cell kept approximately half of the VPD450 fluorescence intensity of its parent cell.

Statistical data analysis was conducted using GraphPad version 9 statistical software. The distribution of the examined variables was examined with Kolmogorov-Smirnov and Shapiro-Wilk normality tests. A significance level of *p* < 0.05 was set for all analyses.

## 5. Conclusions

Our findings demonstrate that intravenous infusion of ketamine induces rapid but transient immunological changes in patients with treatment-resistant depression ex vivo. A single infusion led to transient activation of T cells and modulation of key cytokines, including a sustained reduction in IL-8, an inflammatory marker previously associated with TRD. These results might support the hypothesis that ketamine’s antidepressant effects could be mediated by immunomodulatory mechanisms. However, to accurately determine the direct relationship between ketamine’s immunomodulatory effects and the final clinical response, further and more detailed studies—ideally including measurements throughout the entire 4-week period—should be conducted.

Notably, while ex vivo data showed time-dependent shifts in T-cell activation and cytokine profiles, in vitro effects were limited and dependent on ketamine dose and cellular context. The divergence between ex vivo and in vitro findings suggests that ketamine’s immunomodulating properties likely involve complex systemic pathways rather than direct lymphocyte interaction alone.

The consistent suppression of IL-8 across models highlights its potential as a biomarker for inflammation-related subtypes of depression and response to ketamine therapy. Future research should focus on integrative approaches to better understand ketamine’s neuroimmune mechanisms and identify immunological predictors of treatment response.

## Figures and Tables

**Table 1:** Characteristics of TRD patients receiving ketamine, participating in the ex vivo part of the study.

	TRD (*n* = 18)
Sex (male/female)	5/13
Age (years) ^1^	44.94 ± 19.72
BMI (kg/m^2^) ^1^	28.24 ± 6.27
Current episode length (weeks) ^1^	41.1 ± 33.86
Bipolar depression (male/female)	7 (3/4)
Unipolar depression (male/female)	11 (2/9)
MADRS before ^1^	28.9 ± 8.5
MADRS after ^1^	19.3 ± 12 *
Delta MADRS ^1^	9.6 ± 8.6
Treatment:	
SSRIs	5
SNRIs	7
SARIs	1
NaSSAs	6
TCAs	1
Atypical antipsychotics	11
Antiepileptic drugs	7
Low-potency antipsychotics	3
Lithium	5

TRD—treatment-resistant depression, BMI—body mass index, SSRIs—serotonin reuptake inhibitors, SNRIs—serotonin and norepinephrine reuptake inhibitors, SARIs—serotonin antagonist and reuptake inhibitors, NaSSAs—noradrenergic and specific serotonergic antidepressants, TCAs—tricyclic antidepressants. * *p* < 0.0001 compared to MADRS before, the Wilcoxon test. Please note that some patients received more than one medication. ^1^ Data are presented as mean ± SD.

## Data Availability

The data presented in this study are available upon request from the corresponding author.
